# Bacteria in the blood of healthy stray dogs infested by ticks in northern Mexico

**DOI:** 10.5455/javar.2024.k757

**Published:** 2024-03-31

**Authors:** Fernando Mejía García, Sergio Iván Barraza Guerrero, Cristina García De la Peña, David Ramiro Aguillón Gutiérrez, Quetzaly Karmy Siller Rodríguez, César Alberto Meza Herrera, Felipe Vaca Paniagua, Clara Diaz Velásquez, Aldo De la Cruz Montoya, Luis Manuel Valenzuela Núñez

**Affiliations:** 1 Laboratorio de Bioindicadores, Centro de Investigación y Jardín Etnobiológico, Universidad Autónoma de Coahuila, Viesca, Coahuila, México; 2 Universidad Autónoma Agraria Antonio Narro. Torreón, Coahuila, México; 3 Facultad de Ciencias Biológicas, Universidad Juárez del Estado de Durango, Gómez Palacio, Durango, Mexico; 4 Unidad Regional Universitaria de Zonas Áridas, Universidad Autónoma Chapingo, Bermejillo, Mexico; 5 Laboratorio Nacional en Salud: Diagnóstico Molecular y Efecto Ambiental en Enfermedades Crónico-Degenerativas, Facultad de Estudios Superiores Iztacala, Universidad Nacional Autónoma de México, Tlalnepantla, Mexico

**Keywords:** Bacteria, blood, healthy, stray dogs, ticks

## Abstract

**Objective:**

The objectives of this study were to determine the richness, abundance, and diversity of bacteria in stray dogs (*Canis lupus familiaris*) infested by ticks in Comarca Lagunera, northern Mexico, and to establish their pathogenic and or/zoonotic potential.

**Materials and Methods:**

Blood samples from 12 dogs were collected, and their deoxyribonucleic acid was extracted. The V3-V4 region of the 16S ribosomal ribunocleic acid gene was amplified by polymerase chain reaction. Next-generation sequencing (NGS) was performed on a MiSeq Illumina platform, and the data were analyzed using quantitative insights into microbial ecology.

**Results:**

The operational taxonomic units resulted in 23 phyla, 54 classes, 89 orders, 189 families, 586 genera, and 620 bacterial species; among them, 64 species and/or bacterial genera with pathogenic or zoonotic potential were identified, some of which have been reported in the literature as relevant to public health (*Anaplasma phagocytophilum, Brucella* spp*., Clostridium* spp*., Corynebacterium affermentants, Cutibacterium* spp*., Dietzia* spp*., Ehrlichia canis, Fusobacterium necrophorum, Leptotrichia* spp*., Mycobacterium* spp*., Paracoccus* spp*.,* and *Roseomonas gilardii*).

**Conclusion:**

This research offers relevant information on the prevalence of tick-borne diseases as well as other potential zoonotic diseases in the blood of stray dogs parasitized by ticks in northern Mexico. New molecular biology and massive NGS techniques may play an important role in the study and documentation of bacterial profiles from animals in close proximity to humans.

## Introduction

Interactions between microorganisms and animals have a long evolutionary history and therefore have a marked effect on shaping life on our planet. Recent literature has suggested the existence of a blood microbiota [1] that mostly originates from the intestinal microbiome [2]. The bacterial microbiota is fundamental in different vital processes of vertebrates since it plays a transcendental role in their health and well-being, both directly and indirectly, in their physiology, immune system, nutrition, and metabolic processes through different mechanisms of coexistence, such as commensalism, mutualism, symbiosis, and pathogenicity [3].

Generally, blood has been considered a sterile medium throughout the years. However, recent studies using amplicon sequencing of the 16S rRNA gene have demonstrated the presence of bacterial genomes in the blood of people and animals.

Some bacteria, such as *Anaplasma phagocytophilum, A. marginale, A. platys, Borrelia burgdorferi, Babesia canis, Bartonella* spp*., Coxiella burnetii, E. canis, Ehrlichia chaffeensis, Rickettsia rickettsii, Yersinia pestis,* and *Mycoplasma* spp., have been effectively isolated in different studies from the blood of dogs, with the vectorial activity of different species of ticks being the main cause of these transmissions [4–9]. These studies have been developed mostly with increasingly sensitive, rapid, and specific technologies such as serological and molecular technologies (polymerase chain reaction (PCR) and sequencing) [5]. It is well known that tick-parasitizing dogs are potential transmitters of bacterial diseases that can cause illness in animals and people. The close coexistence of dogs with people encourages ticks to use humans as hosts, becoming a serious public health problem. Knowing the bacteria present in dog blood can contribute to the design of prevention programs for zoonotic bacterial diseases. The information generated in this study contributes to establishing the richness, abundance, and diversity of bacteria in stray dogs in northern Mexico, as well as their pathogenic and/or zoonotic potential.

## Materials and Methods

### Ethical approval

All the methods and activities of this study were in strict accordance with accepted guidelines for the ethical use, care, and welfare of animals in research at international and national levels, with institutional approval reference number UJED-FCB-2018-12.

### Location, experimental sites, and environmental conditions

The study was carried out in four locations in northern Mexico: three in the state of Durango [Bermejillo (25°53’17” N, 103°37’20” W), Gómez Palacio (25°33’40” N, 103°29’54” W), Tlahualilo (26°6’12” N, 103°26’26” W)], and one in the state of Coahuila [Matamoros (25°31’58.8” N, 103°15 ’0” W)]. This region is in the Mexican northeast, formed by the states of Coahuila and Durango (24°22 and 26°52 N, 102°03 and 104°46 W).

### Collection of samples by sex

Sampling was conducted over three months, from September to November 2019. The samples were obtained from stray dogs with at least six ticks on their bodies; the sex of each canine was determined. After selecting the dog, the puncture area was cleaned with cotton and 70% alcohol; blood samples were taken using 3 ml syringes from the cephalic vein. The blood was stored in Vacutainer tubes (EDTA as an anticoagulant), and each tube was labeled with the date and place of collection, as well as the sex of the canine.

Ten drops (approx. 50 mg of blood in wet weight) were collected for each sampled dog and placed in BashingBead™ lysis tubes from the ZymoBiomics Research™ kit, containing 750 µl of lysing/stabilizing solution (Xpedition^TM^). Finally, samples were processed in a Terralyzer (Zymo Research) cell disruptor.

### Extraction, deoxyribonucleic acid (DNA) quantification, and amplification

DNA extraction and visualization were performed following the protocol by Barraza et al. [3,10]. The amplification of the V3-V4 region of the 16S ribosomal ribunocleic acid gene was performed using the primers S-D-Bact-0341-b-S-17, 5’-CCTACGGGNGGCWGCAG-3’ and S-D-Bact-0785-a-A-21, 5’-GACTACHVGGGTATCTAATCC-3’, which produces an amplicon of ~460 bp. PCR was performed following the Illumina protocol for 16S metagenomics, as well as quantification, normalization (equimolarity), library pooling, and massive next-generation sequencing (NGS) (MiSeq Illumina^®^ 2 × 250 paired-end reads).

### Bioinformatics Analysis

Sequences were processed using quantitative insights into microbial ecology v.1.9.0 software. Forward and reverse sequences were assembled based on a quality criterion of Q30. Chimeric sequences were removed, and operational taxonomic units (OTUs) were selected using the UCLUST method at 97% similarity. Taxonomy was assigned using the EzBioCloud database, using a representative sequence for each OTU [11]. The OTU table was built in biom (biological observation matrix) format, and the domains were separated. The absolute abundance of OTUs was determined, and the number of sequences was plotted by the number of taxa at the genus level to observe the coverage depth (asymptote trend curves); PAST Ver. 3.15 software was used. Beta diversity was calculated using the Bray-Curtis index; the obtained matrix was used to perform a PERMANOVA test (*p* < 0.05) to observe significant differences in the microbiota between the sexes of the dogs. In addition, alpha diversity was obtained with the Shannon and Simpson indices; nonparametric Student’s *t* tests (999 Monte Carlo permutations) were used to detect significant differences between sexes.

Relative abundances were obtained for phylum, class, order, family, genus, and species. At the phylum level, a stacked bar graph was generated in *R*, while for family and genus, heatmaps were created (hierarchical clustering method with Euclidean measurement for the dendrogram of the samples) using Morpheus (https://software.broadinstitute.org/morpheus). Finally, an exhaustive literature review was performed to determine potentially pathogenic agents for dogs and humans.

## Results

**Figure 1.**
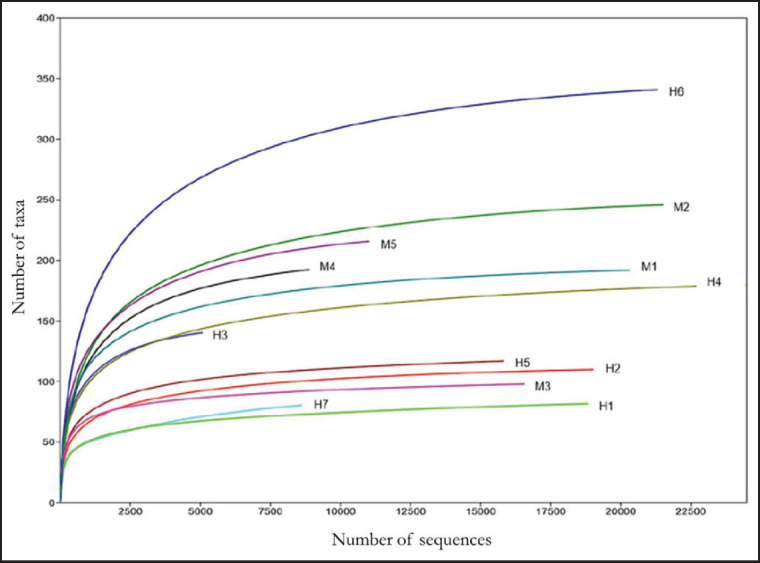
Rarefaction curve sequences showing the blood microbiota cover depth (number of sequences *vs*. taxa number) from males (M) and females (H) *C. lupus familiaris.*

The mean number of bacterial sequences obtained for all samples before assembly was 120,337; the mean number of assembled sequences was 45,252, and the mean number of discarded sequences was 75,085; on average, 2,531 chimeras were eliminated, thus obtaining an average of 42,556 quality sequences. Subsequently, after taxonomic assignment, a mean of 36,321 bacterial sequences was obtained (Table 1). An adequate depth of coverage was recorded since almost all the samples reached the asymptote near 8,500 sequences (Fig. 1).

**Figure 2.**
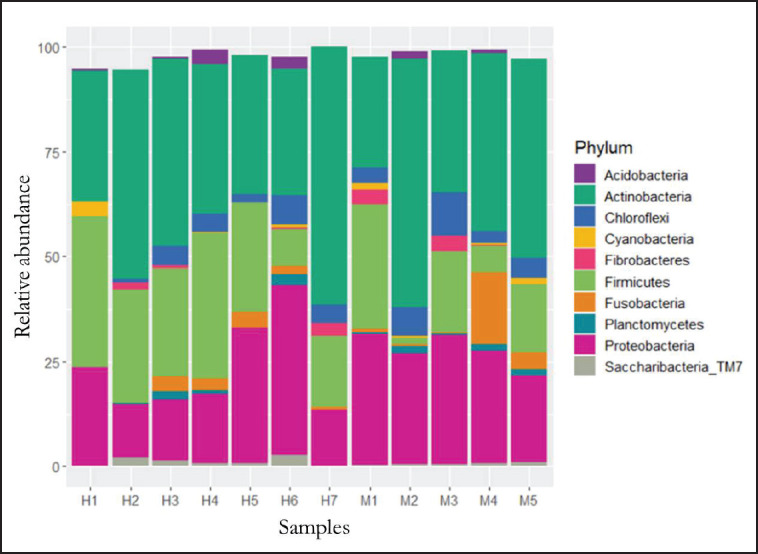
Relative abundance (%) of the bacterial classes founded in the blood of males (M) and females (H) *C. lupus familiaris* (only the ten most abundant phyla are showed).

**Figure 3.**
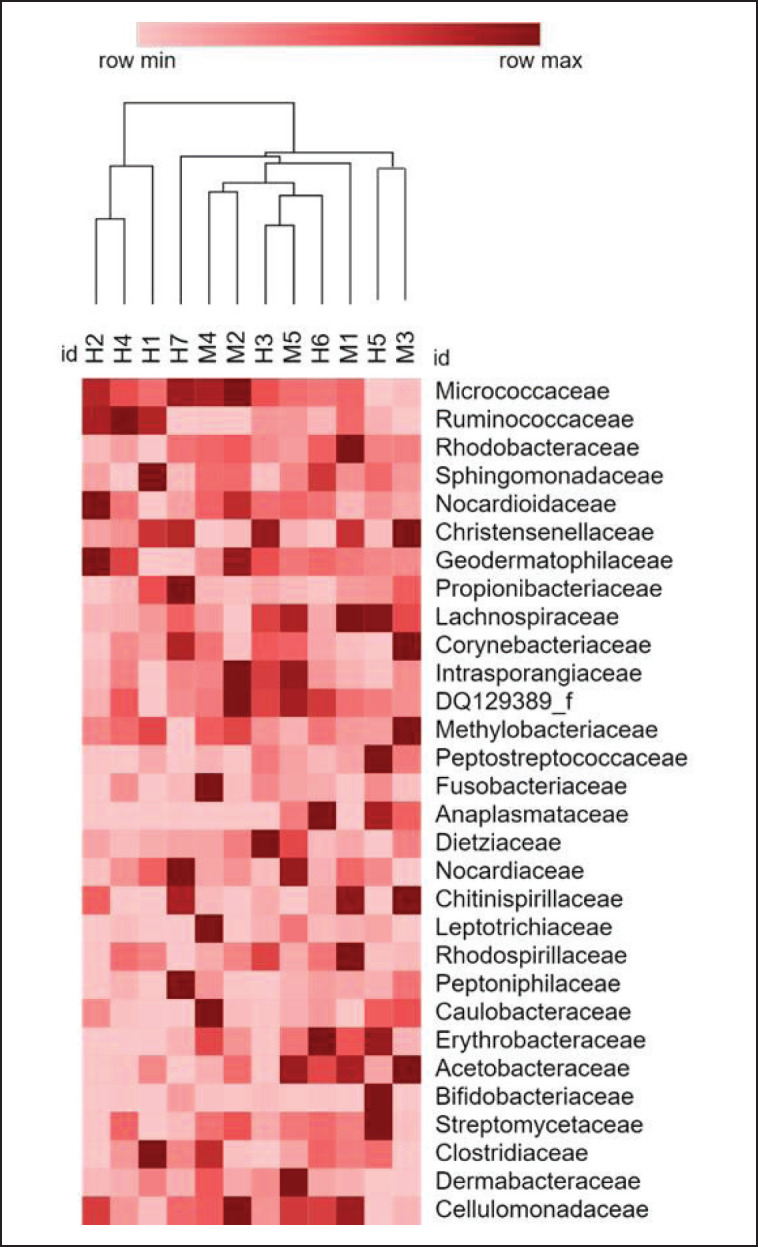
Heatmap of the bacterial families founded in the blood of males (M) and females (H) *C. lupus familiaris,* whose relative abundance was greater than 0.01%.

**Figure 4.**
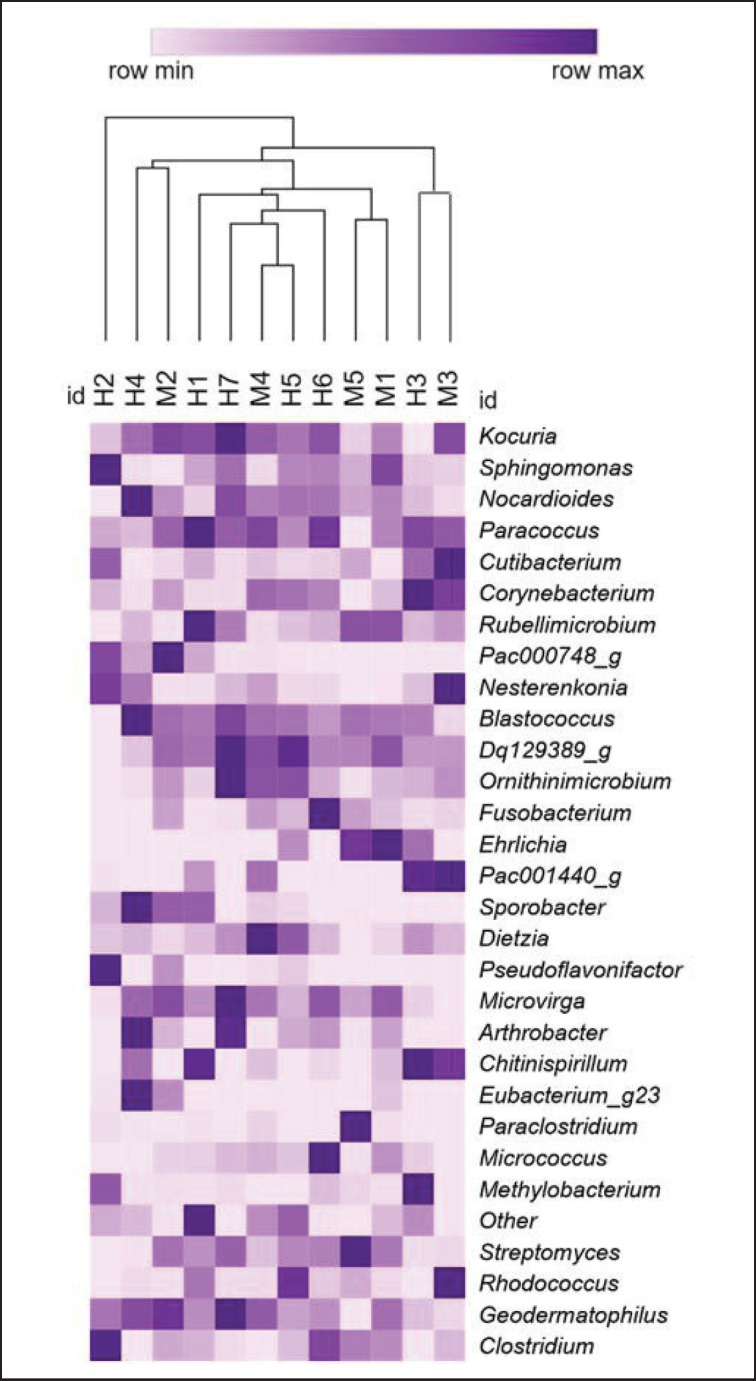
Heatmap of the bacterial genera founded in the blood of males (M) and females (H) *C. lupus familiaris*, whose relative abundance was greater than 0.01%.

No significant difference was found between the bacteria present in the blood of female and male *Canis lupus familiaris* (PERMANOVA: pseudo-F = 0.983, *p* = 0.491). Regarding alpha diversity, the Shannon index mean was 7.92, the Simpson index was 0.98, and no significant difference was observed between sexes for either of the two indices (Shannon: *t* = 1.49 *p* = 0.146; Simpson: *t* = 1.61 *p* = 0.132). A total of 23 phyla were identified, of which Actinobacteria (*x–* = 41%), Proteobacteria (*x–* = 24%) and Firmicutes (*x–* = 20%) were the most abundant (Fig. 2). A total of 54 classes were found, among which Actinobacteria (*x–* = 39%), Alphaproteobacteria (*x–* = 23%) and Clostridia (*x–* = 18%) were the most abundant. A total of 89 orders were obtained, among which Clostridiales (*x–* = 19%) was the most abundant, followed by Micrococcales (*x–* = 17%) and Propionibacteriales (*x–* = 8%). Of the 189 families identified, Micrococcaceae (*x–* = 11%), Ruminococcaceae (*x–* = 9%) and Rhodobacteraceae (*x–* = 6%) were predominant (Fig. 3). A total of 586 genera were identified, with *Kocuria* (*x–* = 6%), *Sphingomonas* (*x–* = 5%) and *Nocardioides* (*x–* = 5%) being the most abundant (Fig. 4). Furthermore, 620 species were recorded. Finally, 64 genera and/or species of bacteria were identified as pathogenic or potentially pathogenic for both people and some animals (Table 2).

**Table 1.** Bacterial sequences obtained from the blood of *C. lupus familiaris*. H = female, M = male, QE = chimeras removed, SC = quality sequences after chimera removal, SB = bacterial sequences after taxonomic assignment.

Sample	Total	Assembled	Discarded	QE	SC	SB
H1	119,388	37,837	81,551	1,595	36,109	30,778
H2	131,546	55,635	75,911	2,470	52,958	45,647
H3	134,309	41,361	92,948	2,766	38,456	29,417
H4	100,708	52,055	48,653	5,021	46,836	42,437
H5	98,540	32,237	66,303	731	31,392	28,421
H6	123,758	57,481	66,277	4,107	53,141	46,313
H7	130,022	29,132	100,890	304	28,713	23,576
M1	132,232	54,805	77,427	3,411	51,175	43,759
M2	122,526	65,452	57,074	4,016	61,219	53,250
M3	149,541	42,182	107,359	605	41,444	32,959
M4	105,658	37,169	68,489	2,480	34,548	29,280
M5	95,820	37683	58,137	2,861	34,682	30,013
Mean	120,337	45,252	75,085	2,531	42,556	36,321

## Discussion

The results obtained in this study represent the first characterization of the bacteria present in the blood of stray dogs (*C. lupus familiaris*) in northern Mexico. Some authors agree that the presence of bacterial DNA in blood is due to physiological translocation, a phenomenon in which live bacteria or their products cross the intestinal barrier, the oral cavity, or the skin of the host into the bloodstream [12]. The bacteria found in the canine blood were mostly Actinobacteria (41.33%), Proteobacteria (24.20%), and Firmicutes (20.64%). These taxa coincide with the results obtained in human studies, where Proteobacteria (80%–87%) and Actinobacteria (6%–10%) are the phyla with the greatest abundance [1,12,13]. On the other hand, studies conducted in healthy and sick dogs show similar results to those obtained in the present research, as the phyla Actinobacteria, Proteobacteria, and Firmicutes are found as part of the blood and gut microbiota of *C. lupus familiaris* [14,15]. The data obtained support the theory that the blood microbiota originates through processes of bacterial translocation from the gastrointestinal tract into the blood; therefore, the blood microbiota is closely related to the diet and gastrointestinal health status of the animal [16].

In the blood of *C. lupus familiaris,* the most abundant classes recorded were Actinobacteria (39%) and Alphaproteobacteria (23%). Bacteria belonging to the class Actinobacteria have been reported as obligate endosymbionts and abundant members of microbial communities, described as controllers of infections in wild animals and livestock, as well as those associated with diseases such as tuberculosis, mycetoma, nocardiosis, allergic pneumonia, and dermatitis [17]. On the other hand, some Alphaproteobacteria have been reported to live inside the cells of many complex life forms, and others act as parasites or beneficial symbionts inside the cells of such organisms [18].

**Table 2.** Prevalence (%) of potentially pathogenic genera/species recorded in the blood of *C. lupus familiaris* according to the available literature.

Genus/species	(%)	Gram	Genus/species	(%)	Gram
*Actinomadura*	75	+	*Haematobacter*	16.6	−
*Agromyces*	8.3	+	*Helcococcus*	16.6	+
*Anaerococcus*	66.6	+	*Janibacter*	58.3	+
*Anaerotruncus*	8.3	−	*kytococcus*	50	+
*Anaplasma*	16.6	−	*Leifsonia*	8.3	+
*Arcobacter*	25	−	*Leptotrichia*	41.6	−
*Atopobium*	8.3	+	*Leucobacter*	66.6	+
*Aureimonas*	33.3	−	*Methylobacterium*	91.6	−
*Bacillus*	50	+	*Microbacterium*	75	+
*Bosea*	25	−	*Micrococci*	75	+
*Brachybacterium*	83.3	+	*Mycobacterium*	75	+
*Brevibacterium*	66.6	+	*Nesterenkonia*	100	+
*Brevundimonas*	41.6	−	*Nocardia*	8.3	+
*Brucella*	16.6	−	*Ochrobactrum*	8.3	−
*Campylobacter*	50	−	*Oerskovia*	16.6	−
*Cellulosimicrobium*	25	+	*Paeniclostridium*	25	+
*Clostridium*	83.3	+	*Pannonibacter*	8.3	−
*Corynebacterium*	100	+	*Paracocci*	100	−
*C. afermentans*	8.30	+	*Parvimonas*	58.3	+
*Cutibacterium*	100	+	*Peptoanaerobacter*	33.3	+
*Dermabacter*	16.6	+	*Peptoniphilus*	75	+
*Dermacoccus*	8.3	+	*Rhodococcus*	83.3	+
*Dietzia*	91.6	+	*Roseomonas*	83.3	−
*Eggerthella*	8.3	+	*R. gilardii*	8.3	−
*Ehrlichia*	33.3	−	*Rothia*	66.6	+
*Filifactor*	41.6	+	*Sneathia*	16.6	−
*Finegoldia*	41.6	+	*Streptobacillus*	33.3	−
*Fusobacterium*	83.3	−	*Terrisporobacter*	41.6	+
*F. necrophorum*	16.6	−	*Tissierella*	33.3	−
*F. nucleatum*	50	−	*Trueperella*	8.3	+
*Gardnerella*	16.6	+/−	*Williamsia*	25	+
*Gordonia*	66.6	+	*Wolinella*	8.3	−

The most abundant bacterial genus in the blood samples of *C. lupus familiaris* was *Kocuria* (6%), which belongs to the phylum Actinobacteria. Chermprapai et al. [19] reported this genus as part of the *C. lupus familiaris* skin microbiome, with an abundance of 5.2% ± 0.6%, which agrees with that found in the *C. lupus familiaris* blood samples obtained in the present study. In addition, the theory of its entry from the skin and mucous membranes into physiological fluids is supported [20]. *Kocuria* was first described as *Micrococcus* spp. in 1995 and was later transferred to a novel genus due to the heterogeneity of micrococcal species indicated by phylogenetic and chemotaxonomic analyses [21]. Mostly, members of the genus *Kocuria* are present as commensal microorganisms; however, several species within the genus *Kocuria* have emerged as important pathogens responsible for various diseases, such as endocarditis, meningitis, cholecystitis, urinary tract infections, catheter-linked bacteremia, peritonitis, and abscesses [20].

The genus *Sphingomonas* was the second most abundant in the blood of *C. lupus familiaris*, with a mean abundance of 5%; it belongs to the phylum Proteobacteria and has been isolated from various sources of contaminated and uncontaminated environments, such as marine water, freshwater, groundwater, wastewater, endophytes, terrestrial habitats, sediment (river and subsoil), rhizosphere, and terrestrial soil [22,23]. The presence of *Sphingomonas* in blood samples of *C. lupus familiaris* may be related to the environmental or habitat conditions with which the object of study is in contact. This genus has been associated with wound infections, peritonitis, bacteremia, meningitis, and septicemia, and it has also been found in hospital dialysis equipment, wounds, blood, stored distilled water, and hospital water supplies [23].

Sixty-four potentially pathogenic bacterial genera were identified in the blood of *C. lupus familiaris* (Table 1), 12 of which have been associated with different species of ticks, including *Anaplasma, Methylobacterium, Bacillus, Microbacterium, Micrococcus, Mycobacterium, Nocardia, Corynebacterium, Rhodococcus, Dietzia, Ehrlichia,* and *Fusobacterium*, some of which have already been described as part of the normal oral, skin, or gastrointestinal microbiota [4–7]. The presence of these organisms in the bloodstream may be due to constant contact of the animal’s oral cavity with exposed wounds, infestation by ectoparasites, or physiological translocation, which would allow the exchange of these organisms into the bloodstream. *Anaplasma* (16.6% prevalence in *C. lupus familiaris* blood samples) and *Ehrlichia* (33.3% prevalence in *C. lupus familiaris* blood samples) are intracellular pathogenic bacterial genera, with some species being zoonotic. These genera belong to the order Rickettsiales and family Anaplasmataceae, which are transmitted by vectors (ticks and fleas) [24].

*Anaplasma phagocytophilum* is the causative agent of different diseases in both humans and different animals, which are known as human granulocytic anaplasmosis (HGA), canine granulocytic anaplasmosis, and equine granulocytic [24]. According to Benavides-Arias and Soler-Tovar [25], HGA is an emerging zoonosis of high interest in public health, and its incidence is increasing in America. It should be noted that the dogs sampled for the present study did not show signs of any disease associated with *Anaplasma* species, even when a prevalence close to 20% was found. The genus *Ehrlichia* was also recorded in blood samples of *C. lupus familiaris* from Comarca Lagunera; all *Ehrlichia* species infect vertebrate hosts and are transmitted by ticks. *Ehrlichia chaffeensis* and *Ehrlichia ewingii* are proven agents of human clinical cases in North America (human monocysstic ehrlichiosis, HME), while *E. canis, E. ruminantium,* and *E. ewingii* are pathogens mainly of veterinary importance (canine granulocytic ehrlichiosis and canine monocytic ehrlichiosis) [25].

Similar to *Anaplasma*, no sampled dog showed symptoms associated with *Ehrlichia*, even though its prevalence was 33.3%. The genera *Corynebacterium, Fusobacterium, Paracoccus,* and *Mycobacterium* were found in the blood of *C. lupus familiaris* with a prevalence greater than 50%; all of these genera are associated with acute or serious diseases in both animals and people. *Corynebacterium afermentans*, *Fusobacterium nucleatum,* and *F. necrophorum* were identified in *C. lupus familiaris* samples, which coincides with the work of Corrales et al. [26], who determined *Corynebacterium* and *Fusobacterium* as part of the normal oral microbiota of stray dogs, the same condition for the dogs used in this work. Abbott et al. [27] associated *Corynebacterium ulcerans* with upper respiratory tract infections and emphasized that dogs can carry the organism without it producing an infection, generating a high zoonotic potential. In people, the species *F. nucleatum* has been associated with bacterial endocarditis, breast cancer proliferation, and metastatic progression [28], and more recently, bacteremia associated with COVID-19 [29]. On the other hand, the species *F. necrophorum* identified in the blood of *C. lupus familiaris* with a prevalence of 16.6% is the cause of Lemierre syndrome, known as the “forgotten disease,” which is defined as septic thrombophlebitis of the vein internal jugular [30].

## Conclusion

This study provides relevant information on the prevalence of tick-borne diseases as well as other potential zoonotic diseases in the blood of stray dogs parasitized by ticks in northern Mexico. The most abundant phyla were Actinobacteria, Proteobacteria, and Firmicutes. In addition, 586 genera were found, with *Kocuria* and *Sphingomonas* being the most abundant. In addition, relevant potentially pathogenic bacteria such as *Anaplasma*, *Brucella, Clostridium, Corynebacterium, Ehrlichia,* and *Mycobacterium* were determined in the blood of *C. lupus familiaris*, which are related to serious public health and veterinary illnesses in the region and the country. New molecular biology and massive NGS techniques may play an important role in the study and documentation of bacterial profiles from animals in close proximity to humans.

## List of Abbreviations

DNA, deoxyribonucleic acid; HGA, human granulocytic anaplasmosis; HME, human monocytic ehrlichiosis; NGS, next generetion sequencing; OTU, operational taxonomic unit; PCR, polymerase chain reaction.
